# Sensing the host atmosphere: carbon dioxide regulates virulence and drug response in medically relevant fungi

**DOI:** 10.4103/mgr.MEDGASRES-D-26-00038

**Published:** 2026-05-19

**Authors:** Thaís P. Mello, Lívia S. Ramos, Marta H. Branquinha, André L.S. Santos

**Affiliations:** 1 Laboratório de Estudos Avançados de Microrganismos Emergentes e Resistentes (LEAMER), Departamento de Microbiologia Geral, Instituto de Microbiologia Paulo de Góes (IMPG), Centro de Ciências da Saúde (CCS), Universidade Federal do Rio de Janeiro (UFRJ), Rio de Janeiro, RJ, Brazil; 2 Rede Micologia RJ, Fundação de Amparo à Pesquisa do Estado do Rio de Janeiro (FAPERJ), Rio de Janeiro, RJ, Brazil

**Keywords:** antifungal resistance, *Aspergillus*, biofilm, *Candida*, carbon dioxide, *Cryptococcus*, host environment, morphogenesis, proliferation, *Scedosporium*

## Abstract

**Facts**

1. Physiological carbon dioxide (CO_2_) levels function as a key environmental cue that allows fungal pathogens to sense and adapt to host conditions, coordinating metabolic and developmental responses.
2. CO_2_ modulates morphogenesis, biofilm formation, capsule production and developmental pathways, directly contributing to fungal pathogenicity and persistence in host tissues.
3. CO_2_-induced alterations in membrane composition, metabolism and stress pathways can influence antifungal susceptibility and tolerance across different species.
4. These regulatory effects are observed in diverse clinically important fungi, highlighting CO_2_ as a conserved and versatile modulator of fungal biology.
5. Insights into CO_2_ signaling provide a more physiologically relevant framework for predicting antifungal efficacy and optimizing therapeutic strategies.

**Open Questions**

1. What is the impact of dynamic CO_2_ gradients within host-associated niches on fungal virulence, fitness and treatment outcomes?
2. How does CO_2_ signaling interact with other environmental cues (e.g., pH, oxygen availability, nutrient status, and microbiota-derived factors) to regulate pathogenicity?
3. To what extent can CO_2_-responsive pathways be safely and effectively exploited as antifungal targets *in vivo*?

Carbon dioxide (CO_2_) is a pivotal environmental signal that influences the physiology, development and pathogenic potential of fungi. Beyond its role as a metabolic by-product, CO_2_ functions as a conserved signaling molecule that enables fungal pathogens to sense and adapt to host niches. This review synthesizes current knowledge on how fluctuations in CO_2_ concentration modulate growth dynamics, morphogenesis, virulence-associated attributes and antifungal susceptibility in clinically important yeasts and filamentous fungi. In *Candida* species, particularly *Candida albicans*, physiological CO_2_ levels encountered in host tissues (~5%) act as a potent morphogenetic signal, driving the yeast-to-hypha transition, promoting biofilm formation and inducing metabolic reprogramming; processes tightly linked to tissue invasion and persistence. CO_2_ exposure also alters membrane composition and stress response pathways, thereby modulating susceptibility to antifungal agents. In *Cryptococcus neoformans*, elevated CO_2_ concentrations promote capsule biosynthesis and enlargement, alongside extensive remodeling of surface architecture and membrane composition. These CO_2_-driven adaptations strengthen resistance to host immune defenses and are associated with increased tolerance to antifungals. In Aspergillus species, CO_2_ availability influences growth, mycotoxin biosynthesis and developmental trajectories by shifting the balance between asexual and sexual reproduction, with downstream effects on virulence and antifungal responsiveness. In *Scedosporium*/*Lomentospora* species, CO_2_ promotes conidial germination and developmental transitions that may indirectly impact antifungal susceptibility and pathogenic fitness. Collectively, these findings highlight environmental CO_2_ as a pivotal and dynamic regulator of fungal biology, offering key insights into host-pathogen interactions, informing the prediction of antifungal susceptibility profiles and supporting the rational optimization of therapeutic strategies in clinical settings.

## Introduction

The ubiquitous gas carbon dioxide (CO_2_) represents the terminal product of the complete oxidative degradation of organic carbon and serves as a primary substrate for photosynthesis, sustaining the global carbon cycle. As a central component of Earth’s atmosphere, CO_2_ plays a pivotal role in maintaining ecological balance, regulating climate dynamics and supporting the metabolic interdependence between autotrophic and heterotrophic organisms, enabling the persistence and diversification of life on our planet.^1^,^2^,^3^ Over the past 150 years, atmospheric CO_2_ concentrations have increased by approximately 50%, rising from preindustrial levels of approximately 280 parts per million (ppm) to more than 400 ppm.^4^ Projections based on contemporary climate models indicate that, depending on future emission trajectories and mitigation efforts, atmospheric CO_2_ levels may reach between 550 and 1000 ppm by the year 2100.^5^,^6^

As expected, CO_2_ exerts a fundamental and multifaceted influence on the physiology, metabolism and ecological distribution of microorganisms on Earth. In fungi, in particular, CO_2_ fulfills a dual and highly integrated role. It is both a metabolic by-product of cellular respiration and a biologically active signaling molecule that enables cells to sense, interpret and adapt to their surrounding environment.^3^,^7^ Through conserved signaling pathways, fluctuations in environmental CO_2_ concentrations regulate gene expression programs that coordinate growth, stress adaptation and developmental decisions. Consequently, fluctuations in CO_2_ levels profoundly influence key fungal processes, including morphogenetic transitions, sporulation, capsule biosynthesis and the development of complex multicellular structures such as biofilms. These CO_2_-driven adaptations, in turn, indirectly modulate fungal susceptibility to environmental stressors, including exposure to antifungal agents, by reshaping cellular architecture, metabolic states and stress response pathways.^3^,^8^,^9^

**Figure 1:**
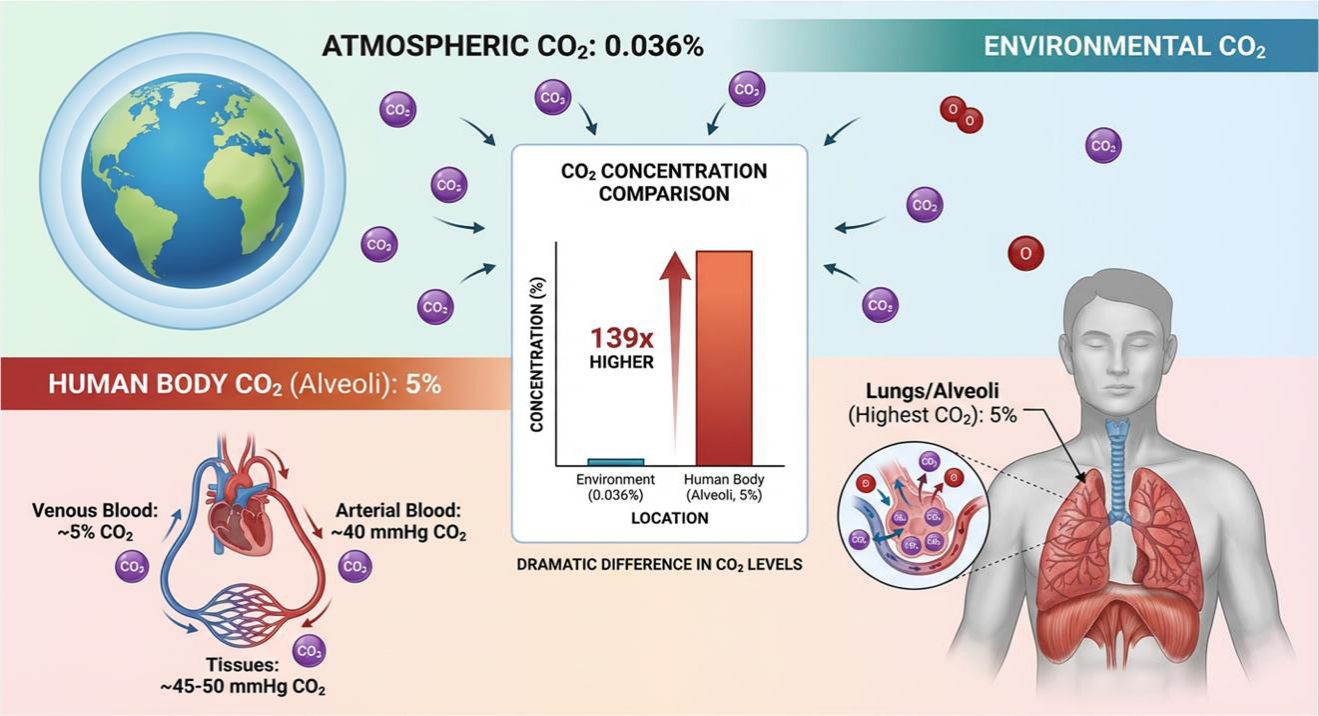
Comparison of CO_2_ concentrations in environmental air (0.036%) versus human body sites (up to 5%). The human body maintains CO_2_ levels approximately 139 times higher than atmospheric concentrations in sites such as alveolar air, venous blood and metabolic tissues. This concentration gradient facilitates respiratory gas exchange and is maintained through continuous cellular respiration and pulmonary ventilation. Created with SciSpace AI (2026). CO_2_: Carbon dioxide.

**Figure 2:**
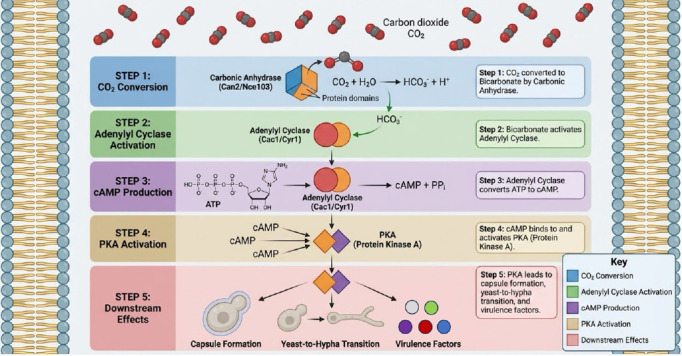
CO_2_ sensing and signaling pathway in fungi. CO_2_ is converted to bicarbonate (HCO_3_^−^) by carbonic anhydrase (Can2/Nce103) (Step 1), which activates adenylyl cyclase (Cac1/Cyr1) (Step 2) to produce cAMP from ATP (Step 3). cAMP then activates PKA (Step 4), leading to downstream cellular responses including capsule formation, yeast-to-hypha morphological transition and the expression of other virulence factors (Step 5). Created with SciSpace AI (2026). cAMP: Cyclic adenosine monophosphate; CO_2_: carbon dioxide; H_2_O: hydrogen oxygen; PKA: protein kinase A; PPi: pyrophosphate.

The CO_2_ sensing capacity of fungi is particularly relevant for species that colonize and infect the human body, where they encounter highly variable CO_2_ levels across different anatomical sites. In blood and specific host niches, CO_2_ concentrations can exceed 5%, while atmospheric levels are approximately 0.036% (**Figure 1**).^10^,^11^,^12^ This CO_2_ gradient allows fungi to activate virulence traits within host tissues, optimizing their pathogenic potential while conserving resources in non-host environments. Fungi have evolved sophisticated molecular machinery to detect and transduce CO_2_ signals into cellular responses. The primary CO_2_-sensing mechanisms identified in fungi involve two key enzymatic systems: carbonic anhydrase and adenylyl cyclase, which work together to convert environmental CO_2_ signals into intracellular signaling cascades (**Figure 2**).^3^,^13^,^14^

The enzyme carbonic anhydrase catalyzes the reversible conversion of CO_2_ into bicarbonate (HCO_3_^−^) and protons, a crucial reaction for both cellular metabolism and signal transduction.^14^ The resulting HCO_3_^−^ acts as a signal that transmits the CO_2_ message (**Figure 2**). Structurally, carbonic anhydrases are classified into the α-, β-, γ- and δ-classes, all of which share a conserved zinc-centered active site typically coordinated by histidine or cysteine residues.^14^ The first fungal carbonic anhydrase characterized was the Nce103 from the yeast *Saccharomyces cerevisiae* (*S. cerevisiae*), and its expression involves the activating transcription factor/cyclic adenosine monophosphate (cAMP) response element binding protein transcription factor Cst6 and the AGC family protein kinase Sch9, a conserved regulatory mechanism across multiple fungal species, including *Candida albicans* (*C. albicans*) and *Candida glabrata* (*C. glabrata*).^12^,^14^ Subsequently, the formed HCO_3_^−^ activates the adenylyl cyclase, establishing a direct link between the external CO_2_ and intracellular signaling.^15^ This activation leads to cAMP synthesis, a second messenger that regulates several physiological processes (**Figure 2**).^14^ In *C. albicans*, this connection is mediated by Cyr1p, an adenylyl cyclase that integrates multiple environmental signals, including CO_2_/HCO_3_^−^ through its C-terminal catalytic domain.^11^ Mutational analyses have revealed that a specific lysine residue at position 1373 (Lys_1373_) is indispensable for the CO_2_/HCO_3_^−^ responsiveness of Cyr1p, effectively gating the enzyme’s activity in response to environmental CO_2_ fluctuations.^11^ When HCO_3_^−^ binds to this receptor site, it activates Cyr1p, leading to increased production of cAMP and subsequent activation of protein kinase A (PKA)-dependent signaling cascades.^11^,^13^ This Ras/cAMP/PKA pathway is fundamental to CO_2_-induced morphological and physiological changes in multiple fungal pathogens (**Figure 2**).^16^

Beyond the adenylyl cyclase/cAMP pathway, fungi possess alternative CO_2_-sensing mechanisms that operate through distinct molecular circuitry. The basic leucine zipper transcription factor Rca1p governs a cAMP-independent CO_2_-response pathway in *C. albicans*, primarily by controlling carbonic anhydrase gene expression.^17^ This transcriptional regulatory system functions in parallel with the Cyr1p-cAMP axis, indicating that fungal CO_2_ sensing employs multiple, potentially redundant pathways that may converge on shared effector programs.^17^ In *S. cerevisiae*, the Rca1p orthologue Cst6p recognizes a conserved DNA sequence motif (TGACGTCA) within carbonic anhydrase promoters, directly linking CO_2_-responsive transcription to enzyme expression levels.^17^

The multi-pathway architecture provides fungi with robust and flexible mechanisms to sense and respond to CO_2_ across a range of concentrations and physiological conditions. Moreover, the evolutionary conservation of CO_2_-sensing mechanisms across fungal species, from model organisms such as *S. cerevisiae* to major human pathogens such as *C. albicans*, underscores the fundamental importance of this signaling pathway in fungal biology.^11^,^17^

## Database Search Strategy

In this narrative review, we included studies addressing the role of CO_2_ in fungal physiology, morphogenesis, virulence and antifungal susceptibility. The majority of the references were full-text articles published in English between approximately 2000 and 2026, encompassing both yeast and filamentous fungal pathogens of global clinical relevance. The literature search was primarily conducted using the PubMed database to identify relevant publications. The following keywords and combinations were applied: (1) carbon dioxide or CO_2_, (2) fungal pathogens, (3) *Candida*, (4) *Cryptococcus*, (5) *Aspergillus*, (6) *Scedosporium*, (7) *Lomentospora*, (8) virulence, (9) morphogenesis, and (10) antifungal susceptibility or resistance. Representative studies were screened with emphasis on host-relevant CO_2_ conditions and mechanistic insights into CO_2_-sensing pathways. Additional relevant articles were identified through manual screening of reference lists to further support and contextualize the discussed topics.

## Carbon Dioxide and *Candida* Species

*C. albicans* is a polymorphic fungal species that asymptomatically colonizes the oral cavity and gastrointestinal tract of most healthy individuals as a component of the normal microbiota, and, in women, the vaginal mucosa. Under conditions that disrupt host immune function or microbiota balance, however, this commensal organism can transition to a pathogenic state, causing disease.^18^ Clinical manifestations most commonly involve mucocutaneous sites, including oral, vulvovaginal and cutaneous infections, but in high-risk individuals, the infection may progress to invasive candidiasis, characterized by candidemia and dissemination to deep organs.^18^ Major predisposing factors include immunosuppression, broad-spectrum antibiotic use, mucosal barrier disruption, metabolic disorders such as diabetes mellitus, hormonal imbalance, indwelling medical devices and prolonged hospitalization.^19^

In addition to *C. albicans*, non-*albicans Candida* species (NACs) have increasingly emerged as important etiological agents of candidiasis, reflecting a notable epidemiological shift in many clinical settings. Species such as *C. glabrata*, *Candida tropicalis* (*C. tropicalis*), *Candida parapsilosis* (*C. parapsilosis*), *Candida krusei*, and, more recently, *Candida haemulonii* and *Candida auris* (*C. auris*) are increasingly reported in both mucosal and invasive infections, especially among hospitalized individuals and patients with compromised immune systems.^20^ Importantly, several NACs exhibit distinct virulence traits and reduced susceptibility to commonly used antifungals, particularly azole drugs, contributing to therapeutic challenges and underscoring the need for accurate species identification and antifungal susceptibility testing.^20^

**Figure 3:**
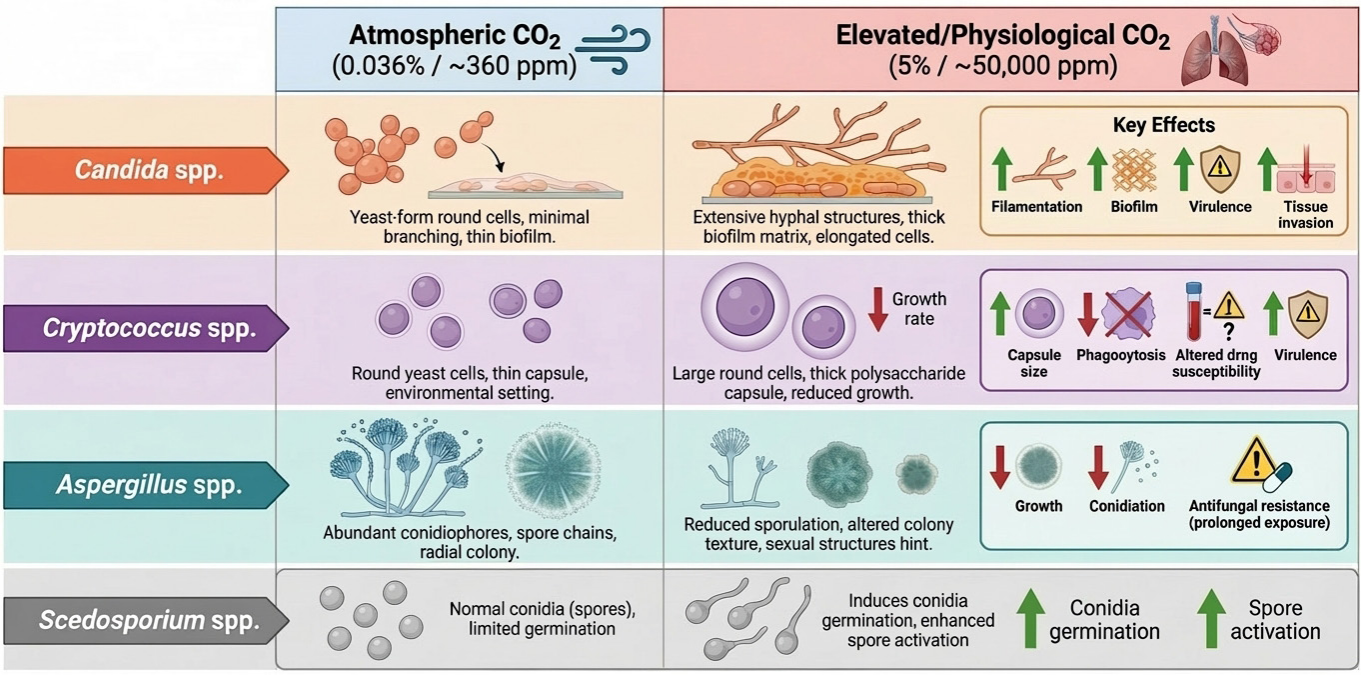
CO_2_-dependent morphological and virulence changes in yeast and filamentous fungi. *Candida* spp. show enhanced filamentation and biofilm formation; *Cryptococcus* spp. exhibit capsule enlargement and altered drug susceptibility; *Aspergillus* spp. display reduced growth and sporulation; *Scedosporium* spp. demonstrate increased conidia germination under elevated CO_2_. ↑: Increase; ↓: decrease; : clinically significant effects; CO_2_: carbon dioxide. Created with SciSpace AI (2026) based on peer-reviewed literature 2000-2026.

The shift of *C. albicans* from a commensal to a pathogenic state is driven by a complex interaction between host- and fungal-derived factors. This process is mainly associated with morphological transitions, in which *C. albicans* cells can undergo reversible transition between yeast, pseudohyphae and filamentous form, which ultimately facilitates the adhesion/invasion of host tissues and the infection development.^11^ In this sense, many host-related environmental cues can influence the morphological transition in *C. albicans*, including temperature, pH, serum, N-acetylglucosamine (GlcNAc), as well as both O_2_ and CO_2_ concentrations.^18^ Within the host body, *C. albicans* encounters a wide range of CO_2_ concentrations, which can vary from approximately 0.03% to 20% or even higher depending on the specific mammalian niche.^21^ For instance, in the intestinal environment, a major habitat of pathogenic *Candida* species, the CO_2_ concentration is around 5–29%, with scarcity of O_2_ and abundance of nitrogen (N_2_).^22^ The ability to modulate morphology in response to environmental CO_2_ fluctuations reflects an adaptive strategy that enables *C. albicans* to thrive in diverse host microenvironments (**Figure 3** and **Table 1**).

**Table 1:** Effects of CO_2_ on physiology and virulence traits in pathogenic fungi

Fungal species	CO_2_ concentration (%)	Morphological effects	Biofilm formation	Virulence traits
*Candida* spp.	5–20	Induce yeast-to-hypha transition	Enhance	↑ Adhesion, invasion
*Cryptococcus neoformans*	5	No major morphological switch	-	↑ Capsule formation
*Aspergillus* spp.	5	Affect germination and hyphal growth	-	↑ Adaptation to host conditions
*Scedosporium* spp.	5	Modulate germination and hyphal development		↑ Adaptation to host conditions

CO_2_: Carbon dioxide.

CO_2_ is a potent inducer of filamentation in *C. albicans*, as extensively demonstrated in the literature.^21^,^23^ Elevated concentrations of CO_2_ directly induce filamentous growth in *C. albicans* via adenylyl cyclase Cyr1, increasing cAMP and activating PKA-dependent hypha-specific genes.^11^ The effect of CO_2_ concentrations in some clinically relevant NACs has also been investigated. In this condition, experiments performed on Lee’s GlcNAc and Lee’s glucose agar demonstrated that incubation under 5% CO_2_ promotes filamentous growth in *Candida dubliniensis* (*C. dubliniensis*) in a strain-dependent manner.^21^ In that study, after 72 hours at 37°C, three of the five isolates evaluated displayed pronounced filamentation when exposed to 5% CO_2_, particularly on Lee’s GlcNAc medium, compared with growth under ambient air CO_2_ conditions. These findings indicate that elevated CO_2_ acts as a morphogenetic stimulus in *C. dubliniensis*, although the magnitude of the response varies among strains.^21^ On the other hand, *C. parapsilosis* isolates were unable to undergo filamentous growth in the same conditions employed for *C. dubliniensis*.^21^ As expected, *C. albicans* isolates used as experimental control, exhibited filamentous growth in both media, showing prominent filamentation in the presence of 5% CO_2_.^21^ In contrast, elevated CO_2_ levels exert an inhibitory effect on filamentation in *C. tropicalis*, suppressing filamentous growth on both Lee’s GlcNAc and Lee’s glucose media, likely through a distinct CO_2_-responsive signaling pathway.^21^ Recent findings have shown that filamentation in *C. tropicalis* is not controlled by the classical cAMP signaling pathway but instead depends on a dedicated CO_2_-sensing mechanism. Within this framework, the conserved carbonic anhydrase and the transcription factor Rca1 function as key sensors of CO_2_ fluctuations and, under certain conditions, act to repress filamentous growth in *C. tropicalis*.^21^

*C. glabrata* has also been reported to undergo morphological changes in response to elevated CO_2_ tension.^24^ Under the experimental conditions evaluated, exposure to 3% CO_2_ for 24, 48 or 72 hours in Sabouraud dextrose broth did not induce pseudohyphal formation. In contrast, limited numbers of filamentous-like cells were detected after 24 hours at 5% and 10% CO_2_. More pronounced morphological alterations were observed at 10% CO_2_ after 72 hours, with cultures exhibiting increased branching and the development of more elongated filamentous forms. Overall, higher CO_2_ concentrations were associated with a reduction in the proportion of budding yeast cells and a concomitant shift toward filamentous growth, indicating a dose- and time-dependent morphogenetic response.^24^ Additionally, the reversibility of filamentous growth was assessed by subculturing the isolates for 1 week in the absence of elevated CO_2_. The findings indicated that *C. glabrata* retained the ability to develop pseudohyphal structures comparable to those observed during the initial filamentation phase.^24^

Interestingly, hyphal development of *C. albicans* inside macrophages occurs independently of CO_2_.^25^ Although the molecular mechanisms underlying hyphal morphogenesis after phagocytosis in *C. albicans* have not been fully elucidated, by using CO_2_-insensitive *C. albicans* mutants, it was demonstrated that hyphal morphogenesis following macrophage engulfment occurs independently of the established CO_2_-sensing pathways.^25^ This was evidenced *in vitro* employing *C. albicans* mutans deficient in enzymatic components of the CO_2_-sensing pathway (*nce103Δ* and *cyr1*^L1373A^) with J774.A1 macrophages. The mutant fungal strains were able to form hyphae like the parent strain (SC5314) with respect to both the temporal progression of cell elongation and the frequency of germ tube formation, as observed using time-lapse microscopy, indicating that these pathways are not involved in hyphal formation.^25^

Within host niches and on abiotic medical devices, *C. albicans* develops dense, structured biofilms in which metabolically generated CO_2_ accumulates and acts as an intra-colony signaling molecule. This locally elevated CO_2_ environment promotes the yeast-to-hypha transition, a critical morphogenetic switch that enhances tissue invasion, biofilm maturation and overall pathogenic potential. This CO_2_-mediated communication occurs through activation of the adenylyl cyclase Cyr1p, a multi-sensor protein that integrates environmental signals such as serum and bacterial peptidoglycan. Importantly, Lys_1373_ was identified as essential for CO_2_/HCO_3_^−^ sensing and regulation of Cyr1p activity. Disruption of this receptor site selectively impairs filamentation within fungal biomasses, highlighting its specific role in structured communities. *In vivo* studies using *Drosophila melanogaster* and murine models of disseminated candidiasis further suggest that metabolic CO_2_ sensing contributes to early colonization and epithelial invasion. Cooperatively, these findings describe a gaseous signaling pathway in *C. albicans* and uncover its molecular basis, supporting the existence of an evolutionarily conserved CO_2_-responsive regulatory system.^11^

**Figure 4:**
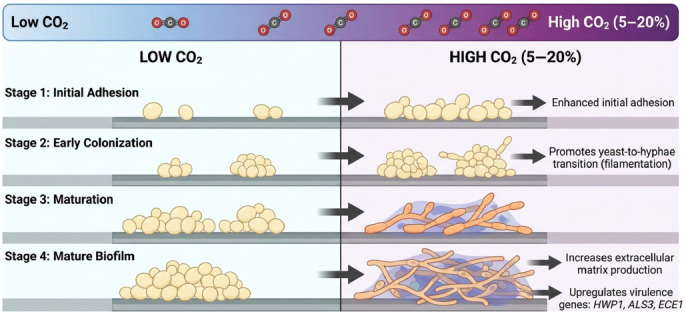
Influence of CO_2_ concentration on *Candida* biofilm formation. There are four sequential stages of biofilm development: initial adhesion, early colonization, maturation and mature biofilm, which illustrates the fully developed biofilm structure under high CO_2_. This structure is characterized by increased extracellular matrix production and upregulation of virulence-associated genes, including *HWP1*, *ALS3* and *ECE1*. CO_2_: Carbon dioxide.

CO_2_ concentration directly influences biofilm development in *C. albicans* (**Figure 4**). Biofilms are highly organized, surface-associated microbial communities in which cells adhere to one another and/or to biotic or abiotic substrates, becoming embedded within a self-produced extracellular matrix. This extracellular matrix, composed of polysaccharides, (glyco)proteins, (glyco)lipids and extracellular DNA, provides structural integrity and protection, contributing to enhanced resistance to environmental stressors and antifungal agents.^26^ Biofilms are considered the preferred mode of life of microorganisms, including fungi, being found both in nature and associated with infections. Microbial cells within biofilms exhibit phenotypic differences in comparison with their planktonic counterparts, including enhanced antimicrobial resistance and virulence. Both *C. albicans* and NACs readily colonize a wide range of indwelling medical devices, including intravascular catheters, urinary catheters, prosthetic valves, joint prostheses and other implanted materials. Their capacity to adhere to these abiotic surfaces and establish mature biofilms is a major contributor to the development of device-associated infections, which are notoriously difficult to eradicate. Biofilm-embedded cells exhibit profound phenotypic changes, including reduced growth rates, metabolic heterogeneity and increased tolerance to either antifungal agents or host immune defenses. As a result, biofilm-associated candidiasis often requires device removal in addition to systemic antifungal therapy. Importantly, it is estimated that up to 80% of all microbial infections are directly or indirectly linked to biofilm formation, underscoring the substantial clinical burden imposed by these structured microbial communities.^26^

Corroborating the aforementioned observations, *C. albicans* biofilms formed on silicone surfaces under elevated CO_2_ conditions (5%) exhibited significantly increased metabolic activity after 24 hours of incubation compared with those grown under atmospheric CO_2_ levels (~0.03%). However, this enhancement was transient, as no significant differences were detected between the two conditions after 48 hours. Despite similar metabolic readings at 48 hours, biofilms formed under 5% CO_2_ displayed greater biomass and thickness at both time points, as confirmed by confocal microscopy and dry weight measurements. These effects were specific to biofilm growth, since planktonic growth rates were not affected by CO_2_ levels.^27^ Regarding the phases of biofilm formation, elevated CO_2_ (5%) significantly enhanced the initial attachment of *C. albicans* cells to silicone compared to atmospheric CO_2_, increasing both the number of attached cells and the formation of larger cell aggregates. This effect suggests improved cell-substrate and cell-cell interactions under high CO_2_. The enhanced aggregation was not due to cell wall stress, as fungal cells showed similar sensitivity to the cell wall stressors Congo red and Calcofluor white under both CO_2_ conditions.^27^ Confocal microscopy revealed that elevated CO_2_ (5%) accelerates *C. albicans* biofilm maturation. At 6 hours, biofilms grown under high CO_2_ showed increased hyphal formation, greater density and early structural organization compared to those grown in atmospheric CO_2_, which were mainly in the yeast form. By 24 hours, biofilms under 5% CO_2_ exhibited a fully mature architecture with organized hyphal structures, whereas those in 0.03% CO_2_ were still maturing. At 48 hours, biofilms in both conditions were mature, but those grown in elevated CO_2_ appeared larger.^27^ Finally, elevated CO_2_ increased cell dispersal from mature *C. albicans* biofilms, resulting in approximately fourfold more released cells compared to atmospheric CO_2_. Therefore, physiological concentrations of CO_2_ enhanced every stage of *C. albicans* biofilm formation on silicon surfaces, from initial attachment through maturation to dispersion, in comparison with atmospheric air CO_2_ concentrations.^27^ Interestingly, exposure to high CO_2_ levels enhanced the capacity of *C. albicans* biofilm to acquire iron and glucose, two key nutrients required for proliferation and biofilm development. Moreover, increased azole resistance was observed in *C. albicans* biofilms formed under 5% CO_2_; however, the mechanisms underlying this resistance have not been elucidated.^27^ Increased environmental CO_2_ concentrations enhance biofilm formation by activating the Ras1–Cyr1–PKA signaling pathway, similarly to its previously characterized role in controlling hyphal morphogenesis in planktonic cells.^27^

White-to-opaque switching in *C. albicans* is a reversible and heritable epigenetic program in which cells alternate between two phenotypically distinct states (white and opaque), each defined by unique cellular morphology, gene expression profiles, mating competence, metabolic preferences and interactions with the host. White cells are round, possess smooth cell wall, form smooth colonies and are optimized for dissemination and interaction with the host immune system, whereas opaque cells are elongated, possess pimped cell wall, form flatter colonies and are specialized for mating competence.^28^ In this regard, the white-to-opaque switching is a prerequisite for sexual mating in *C. albicans* and *C. tropicalis*.^29^ This switching is regulated by a complex transcriptional network influenced by environmental cues such as temperature, CO_2_ levels, pH and nutrient availability (e.g., glucose levels, GlcNAc, carbon source type and nitrogen availability), allowing these fungi to adapt to different host niches and enhance their survival, virulence and evolutionary fitness.^23^ Besides *C. albicans* and *C. tropicalis*, other pathogenic NACs, such as *C. dubliniensis*, also exhibit the white-to-opaque switching, while *C. glabrata* and *C. parapsilosis* do not seem to undergo this phenomenon.^23^

The chitin monomer GlcNAc is widely distributed in the environment and promotes the induction of the opaque phenotype and filamentous growth in *C. albicans*, mainly via activation of the cAMP signaling pathway.^30^ Additionally, high levels of CO_2_ promote white-to-opaque switching in *C. albicans*. Accordingly, incubation of agar cultures of white cells in an atmosphere containing 5% CO_2_ resulted in a 4–16-fold increase in the frequency of opaque and sectored colonies compared with ambient air (~0.03% CO_2_). Notably, the switching frequency increased to over 90% when cultures were incubated in air containing 20% CO_2_.^30^ Additionally, both in the presence and absence of O_2_, high CO_2_ levels induced *C. albicans* switching similarly, indicating that elevated CO_2_ levels, rather than hypoxia, are responsible for white-to-opaque switching in *C. albicans*.^30^ Moreover, the opaque state is maintained at 37°C, indicating that elevated CO_2_ levels within the host promote and stabilize the opaque phenotype, thereby enhancing mating efficiency.^30^ On the other hand, when opaque cells are transferred *in vitro* to 37°C, the temperature of their animal host, they undergo an extensive switch to the white state, suggesting that their primary niche may not be favorable for mating. On the other hand, the opposite pattern is observed in *C. tropicalis*. Although in this species, GlcNAc also acts as an inducer of white-to-opaque switching, as observed in *C. albicans*, elevated CO_2_ levels exhibit the reverse effect in *C. tropicalis*.^29^ In this condition, the switching frequency of *C. tropicalis* in Lee’s GlcNAc medium was 40% under air conditions, whereas in the presence of 5% and 20% CO_2_, it decreased to 0.5–5%, whereas in Lee’s glucose medium, the switching frequency of *C. tropicalis* was below 0.5% under all conditions tested (air, 5% and 20% CO_2_).^29^ Overall, environmental control of white-to-opaque switching differs between *C. tropicalis* and *C. albicans* and can be summarized as follows: in *C. tropicalis*, alkaline pH and GlcNAc favor the induction of the opaque phenotype, whereas acidic pH and high CO_2_ concentrations inhibit this state. In contrast, in *C. albicans*, acidic pH, elevated CO_2_ levels and GlcNAc stimulate the opaque phenotype, while alkaline pH acts as a repressive condition.^29^

Regarding antifungal susceptibility, CO_2_ levels modulate *Candida* responses, with the extent and direction of this effect depending on the species, antifungal class and underlying CO_2_-sensing mechanisms. For instance, exposure to 5% CO_2_ increases the susceptibility of *C. albicans* to echinocandins, resulting in an approximately 8-fold reduction in caspofungin minimum inhibitory concentration (MIC) values compared with ambient air conditions, through Ptc2 (a type 2C protein phosphatase)-mediated Hsp90 dephosphorylation in condensates.^31^ Similar results were observed with micafungin and anidulafungin.^31^ On the other hand, CO_2_ levels did not impact *C. albicans* susceptibility to fluconazole and amphotericin B.^31^ Moreover, physiological CO_2_ levels in the host enhance echinocandin efficacy in a murine model of systemic candidiasis with *C. albicans*, thereby promoting fungal clearance during treatment. Notably, repeated passages of *C. albicans* in mice undergoing caspofungin treatment promote the rapid emergence of *in vivo* drug tolerance, leading to therapeutic failure. These adapted strains exhibit increased resistance to caspofungin under host-level CO_2_ conditions while remaining susceptible under ambient air.^31^

In *C. auris*, however, exposure to 5.5% CO_2_ enhanced the resistance of various isolates to amphotericin B through a CO_2_-sensing pathway involving Rca1/Efg1-Nce103. Rather than merely altering pH, this pathway acts as an active signal that enhances mitochondrial energy production, stabilizes ROS levels and promotes both cell wall and membrane remodeling, collectively increasing antifungal resistance.^32^ On the skin, bacterial colonizers (e.g., *Proteus* and *Klebsiella*) release CO_2_ via urease activity, which fuels the CO_2_-sensing pathway, thereby enhancing both skin tropism and amphotericin B resistance in *C. auris*.^32^

In summary, CO_2_ is a critical environmental signal for *Candida* species, modulating morphology, biofilm formation and pathogenicity. In *C. albicans*, elevated CO_2_ levels stimulate the yeast-to-hypha transition and support host colonization and tissue invasion. Notably, the response to CO_2_ is species-dependent, as some NACs exhibit distinct or even reduced filamentation under high CO_2_ conditions.

## Carbon Dioxide and *Cryptococcus* Species

The genus *Cryptococcus* encompasses globally distributed opportunistic yeasts capable of causing severe and potentially life-threatening infections, particularly in immunocompromised individuals.^33^ These organisms are predominantly environmental, inhabiting soil, decaying wood and avian excreta, with human infection typically initiated by inhalation of airborne propagules. Among them, *Cryptococcus neoformans* (*C. neoformans*) and *Cryptococcus gattii* (*C. gattii*) are the principal etiological agents of cryptococcosis, a disease that generally begins in the lungs and may disseminate to the central nervous system.^33^ Pathogenicity is largely attributed to key virulence factors such as the polysaccharide capsule, laccase and its product, melanin production, the enzymes urease and phospholipase, together with a sophisticated ability to sense and adapt to host-specific environmental conditions.^34^ These features support persistence, immune evasion, resistance to oxidative and other host-imposed stresses and ultimately disease progression. Although these species naturally occupy niches characterized by atmospheric CO_2_ concentrations, inhalation into the mammalian host abruptly exposes them to elevated CO_2_ levels (~5%). As previously noted, this environmental shift functions as a critical host-derived signal, activating adaptive pathways that promote survival, proliferation and successful colonization of host tissues.^35^ In this condition, competitive growth assays have shown that clinical isolates of *C. neoformans* are generally more tolerant to host-relevant CO_2_ levels than environmental strains.^36^,^37^ Moreover, CO_2_-intolerant environmental isolates display attenuated virulence when compared to CO_2_-tolerant counterparts.^36^ Notably, a CO_2_-sensitive strain was able to adapt to elevated CO_2_ concentrations within 21 days in a murine infection model, suggesting that environmental isolates can undergo rapid *in vivo* adaptation and evolutionary selection toward enhanced CO_2_ tolerance.^38^ Similarly to other fungi, CO_2_ sensing in *Cryptococcus* is primarily mediated by carbonic anhydrases, leading to the activation of the cAMP–PKA signaling pathway. This central regulatory cascade integrates CO_2_ availability with other environmental cues to fine-tune cellular growth, metabolic adaptation and the expression of key virulence-associated traits.^39^ Disruption of CO_2_ sensing or signaling pathways impairs fungal fitness under host-like conditions and attenuates virulence in animal models, underscoring the biological relevance of CO_2_ as a regulatory signal during infection.^10^

In *Cryptococcus*, the capsule plays a pivotal role in immune evasion and persistence within the host by inhibiting macrophage-mediated phagocytosis, impairing antigen presentation and modulating cytokine responses. Furthermore, capsular polysaccharides can be actively shed into the extracellular environment, where they exert potent immunomodulatory effects, contribute to tissue damage and facilitate fungal dissemination. The capsule also confers protection against oxidative stress, desiccation and host-derived antimicrobial factors, thereby enhancing fungal survival in hostile host niches.^40^ Notably, host-like concentrations of CO_2_/HCO_3_^−^ within the physiological range rapidly induce enlargement of the polysaccharide capsule in *C. neoformans*, with detectable increases occurring within approximately 3 hours and capsule content per cell scaling proportionally with CO_2_/HCO_3_^−^ levels.^41^ Elevated CO_2_/HCO_3_^−^ also promotes the release of capsular polysaccharide into the surrounding medium and is accompanied by reduced growth rates, extending the cell generation time from 2 to 6 hours, consistent with the high metabolic cost of capsule biosynthesis.^41^ In addition, iron limitation acts synergistically with dissolved CO_2_ to further enhance capsular polysaccharide production and heavily encapsulated cells observed in infected mouse tissues closely reflect these combined conditions.^42^

Studies across multiple clinical isolates of *C. neoformans* have shown that high CO_2_ levels in tissue-culture media or phosphate-buffered saline consistently increase capsule size, although the extent of this response depends on both the strain and the growth medium.^41^,^43^,^44^ A more recent analysis of environmental signals demonstrate that capsule induction in tissue-culture media requires either elevated CO_2_ or exogenous cAMP, whereas nutrient-rich media inhibit capsule expansion even in the presence of CO_2_.^44^ Functionally, CO_2_-induced capsule enlargement reduces complement-mediated phagocytosis by macrophages and is closely associated with enhanced virulence in animal models.^41^,^43^

Interestingly, growth under host-like CO_2_ conditions has been associated with altered responses to antifungal agents in *Cryptococcus*. For instance, in *C. neoformans*, increasing CO_2_ levels from ambient air to host-like concentrations markedly enhances susceptibility to azole antifungals. Under these conditions, the MIC of fluconazole decreased approximately 8- to 10-fold (from 4 to 0.5 μg/mL), and a similar reduction is observed for itraconazole and voriconazole, indicating that this effect is not restricted to fluconazole.^36^ Mechanistically, elevated CO_2_ is thought to alter membrane composition and cellular stress responses, thereby facilitating antifungal drug activity.^36^ Additionally, host-relevant CO_2_ concentrations (5–10%) enhance flucytosine efficacy in *C. neoformans*, with most of the clinical isolates exhibiting 2- to 64-fold decreases in MIC values in a CO_2_-dependent manner.^45^ Transcriptomic analyses and qPCR demonstrate that elevated CO_2_ induces upregulation of the cytosine permease gene *FCY2*, leading to increased uptake of flucytosine. Remarkably, susceptibility to fluorouracil is not altered, indicating that enhanced drug transport, rather than downstream metabolism, is the primary mechanism underlying this effect.^45^ On the other hand, in multiple *C. neoformans* strains, amphotericin B MIC values are essentially unchanged (≤ 2-fold) between ambient air and 5% CO_2_.^36^

Biofilm development in *Cryptococcus* appears to be predominantly an environmental trait favored by external conditions, including low CO_2_, whereas host-like CO_2_ levels inhibit biofilm formation and promote a planktonic lifestyle.^46^ Consistent with this view, a comparison of environmental and host-like conditions showed that *C. neoformans* readily formed biofilms in Dulbecco’s modified Eagle medium at neutral pH under atmospheric CO_2_ levels, while no detectable biofilm formation was observed at either neutral or acidic pH when cultures were incubated at 5% CO_2_. When pH was fixed at 4.0 and CO_2_ concentration was the only variable, biofilms developed exclusively under ambient CO_2_, demonstrating that CO_2_ itself, rather than pH alone, plays a decisive role in regulating biofilm formation.^46^

In contrast to the stimulatory effects observed in *C. albicans*, elevated environmental CO_2_ concentrations suppress mating in *C. neoformans*, indicating that CO_2_ exerts species-specific regulatory effects on fungal developmental programs.^10^ This effect has been associated with increased HCO_3_^−^ generation mediated by the carbonic anhydrase Can2, which suppresses mating induction. In *Can2*-deficient mutants, HCO_3_^−^ was produced only through the spontaneous conversion of CO_2_, a level sufficient to sustain cell viability but insufficient to block mating.^10^ Additionally, Can2-dependent HCO_3_^−^ production was required for basidia and basidiospore formation.^10^ Comparable findings have also been reported for *C. gattii*.^8^

Overall, the evidence indicates that CO_2_ acts as an important environmental signal for *Cryptococcus*, modulating key aspects of its physiology and pathogenicity (**Figure 3**). Elevated CO_2_ levels, like those encountered within the host, influence fungal growth, the expression of major virulence factors, such as capsule formation and adaptation to *in vivo* conditions. Therefore, the ability to sense and respond to CO_2_ is crucial for *Cryptococcus* survival in the host and for the establishment of infection.

## Carbon Dioxide and *Aspergillus* Species

*Aspergillus* species occupy diverse ecological niches where they encounter CO_2_ concentrations ranging from ambient atmospheric levels to the highly elevated concentrations characteristic of fermentation systems, grain storage facilities, infected host tissues and soil microenvironments.^47^ The physiological responses to these varying CO_2_ regimes differ markedly among species, reflecting adaptations to distinct ecological conditions. Model genetic organisms like *Aspergillus nidulans* (*A. nidulans*) exhibit pronounced CO_2_-dependent sexual development, while the species widely used in industry, *Aspergillus niger* (*A. niger*) demonstrates growth phase-specific sensitivity to dissolved CO_2_, and opportunistic pathogens, including *Aspergillus fumigatus* (*A. fumigatus*), undergo morphological shifts and altered drug susceptibility in high-CO_2_ host environments (**Figure 3**).^48^,^49^,^50^,^51^ In pathogenic conditions, elevated tissue CO_2_ concentrations not only alter *A. fumigatus* hyphal architecture and conidiation, but also dramatically reduce antifungal drug efficacy, with MIC values for azoles and polyenes increasing 8- to 128-fold under 12% CO_2_ compared to atmospheric conditions.^51^

Sexual reproduction in *A. nidulans* provides a striking example of CO_2_-dependent development in fungi. While vegetative growth proceeds under ambient atmospheric conditions, sexual differentiation requires elevated CO_2_ concentrations, with sensitivity to CO_2_ limitation varying dramatically across developmental stages.^48^ The fungus proves most vulnerable to CO_2_ depletion during the transition from vegetative to reproductive growth, suggesting that elevated CO_2_ may function as an environmental checkpoint that prevents premature reproductive commitment under suboptimal conditions. The molecular basis for this CO_2_ dependence involves cell wall remodeling pathways, particularly those controlling α-1,3 glucan metabolism. Zonneveld^48^ established that CO_2_ limitation during late vegetative growth simultaneously suppresses α-1,3 glucanase activity and blocks sexual structure formation. Intriguingly, supplementing CO_2_-depleted cultures with alternative carbon sources partially restores sexual differentiation, indicating that CO_2_ may influence development through its role in central carbon metabolism rather than acting solely as a signaling molecule. This coupling of environmental CO_2_ sensing to cell wall biosynthesis provides an elegant mechanism by which fungi can coordinate developmental transitions with nutrient availability and metabolic capacity.

Paradoxically, while elevated CO_2_ promotes sexual development in *A. nidulans*, it generally inhibits vegetative growth across *Aspergillus* species. However, the magnitude of this inhibition depends critically on culture conditions and physiological state. The inhibitory effects of elevated dissolved CO_2_ on *A. niger* vary substantially with culture mode and growth phase.^49^ Batch fermentations prove more susceptible to CO_2_-mediated growth suppression than continuous cultures, while actively dividing cells during lag phase demonstrate the greatest sensitivity.^49^ These growth-suppressive properties have found practical application in food preservation, where modified atmosphere storage leverages CO_2_’s fungistatic effects to prevent spoilage. Complete *Aspergillus* growth suppression occurs in grain stored under 5 g CO_2_ per kilogram at moderate temperatures (25–31°C), while high-CO_2_ packaging (80%) extends the shelf life of bakery products by delaying mold colonization.^52^,^53^

Beyond effects on growth and development, CO_2_ concentration profoundly influences secondary metabolism in toxigenic species, with important implications for food safety. Aflatoxin biosynthesis in *Aspergillus flavus* responds to CO_2_ levels in a context-dependent manner, with environmental factors such as temperature and water activity modulating the magnitude of CO_2_ effects.^54^,^55^,^56^ Across diverse conditions, elevated CO_2_ generally suppresses aflatoxin B1 accumulation during maize kernel infection and in culture, suggesting potential for CO_2_-based interventions to reduce mycotoxin contamination in stored commodities. The mechanism underlying this suppression remains unclear but may involve metabolic shifts that redirect carbon flux away from secondary metabolite biosynthesis under elevated CO_2_.

Despite decades of research documenting CO_2_’s effects on *Aspergillus* growth, development, morphology, drug resistance and secondary metabolism, the underlying sensing mechanisms remain largely unknown. Identifying the molecular components of fungal CO_2_ sensing pathways would enable rational manipulation of these responses for applications ranging from improved antifungal therapies to optimized industrial fermentations and enhanced food preservation strategies.

## Carbon Dioxide and *Scedosporium/Lomentospora* Species

Fungal species belonging to the genera *Scedosporium* and *Lomentospora* represent an emerging group of opportunistic molds of increasing clinical concern, particularly affecting immunocompromised individuals (e.g., patients with leukemia, prolonged neutropenia, or solid organ transplantation), as well as patients with chronic lung diseases (e.g., cystic fibrosis, chronic obstructive pulmonary disease, and bronchiectasis). Additional predisposing factors include traumatic inoculation, surgical procedures, near-drowning events, and extensive environmental exposure to contaminated soil or water.^57^ From a clinical and morphological perspective, these filamentous fungi share similarities with *Aspergillus* species, including hyaline septate hyphae in tissue, which may complicate laboratory diagnosis and delay appropriate therapy.^58^ The genus *Scedosporium* comprises several clinically relevant species, notably *Scedosporium apiospermum* (*S. apiospermum*), *Scedosporium aurantiacum* (*S. aurantiacum*), *Scedosporium boydii* and *Scedosporium minutisporum* (*S. minutisporum*), as well as the closely related *Lomentospora prolificans* (*L. prolificans* formerly *Scedosporium prolificans*). The latter is particularly notorious for its intrinsic multidrug resistance, posing major therapeutic challenges and being associated with high mortality rates in disseminated infections.^55^ These fungi are widely distributed in the environment, frequently isolated from soil, polluted waters and anthropogenic habitats and display remarkable physiological adaptability to diverse environmental stresses. Their capacity to tolerate and respond to fluctuations in atmospheric conditions, including variations in CO_2_ levels, reflects a high degree of metabolic and regulatory flexibility.^59^ A deeper understanding of how environmental cues such as CO_2_ influence growth, development and antifungal susceptibility in these organisms is essential for improving diagnostic accuracy and expanding therapeutic strategies against these challenging pathogens.

Current knowledge regarding the effects of CO_2_ on *Scedosporium*/*Lomentospora* species remains limited, with most evidence pointing to its role in promoting conidial germination under elevated concentrations. Exposure to an atmosphere containing 5% CO_2_ resulted in moderate germination efficiency in *S. minutisporum*, with approximately 75% of conidia germinating within 4 hours. In contrast, *S. apiospermum*, *S. aurantiacum* and *L. prolificans* exhibited markedly higher germination rates, with more than 90% of conidia initiating germ tube formation within the same period.^59^ These enhanced germination efficiencies represent a substantial increase compared with ambient air conditions, indicating that host-relevant CO_2_ levels may act as an environmental cue that stimulates spore activation. As conidial germination constitutes a crucial early step in fungal colonization and tissue invasion, the CO_2_-driven acceleration of this process may facilitate host adaptation, promote successful establishment of infection, and ultimately contribute to the pathogenic potential of these molds in mammalian hosts.

In addition to the enhanced germination rates observed under elevated atmospheric CO_2_ concentrations, a marked upregulation of mitochondrial metabolic activity has also been reported, indicating that CO_2_ exposure may act as a metabolic trigger during the transition from dormant conidia to actively growing hyphal forms.^59^ This metabolic activation is consistent with the high energetic demands associated with isotropic growth, germ tube emergence and early hyphal elongation. The positive correlation between CO_2_ exposure and mitochondrial stimulation supports the notion that CO_2_ functions not merely as a permissive environmental factor but as a regulatory signal capable of promoting metabolic reprogramming. Nevertheless, the specific molecular mechanisms and signaling pathways linking CO_2_ sensing to mitochondrial activation in these species remain to be elucidated and warrant further investigation. Furthermore, the accelerated conidial germination rates resulting from elevated CO_2_ environmental concentrations may have significant indirect consequences for antifungal drug efficacy. The morphological transformation from conidial to hyphal growth phases correlates with altered antifungal drug sensitivity profiles. Specifically, mature hyphal structures of *S. apiospermum*, *S. aurantiacum*, *S. minutisporum* and *L. prolificans* demonstrate increased resistance compared to their conidial counterparts when exposed to triazole antifungals such as fluconazole and voriconazole.^59^ Furthermore, the extending conidial germinations are capable of penetrating the plasma membrane of various host cells, including laryngeal epithelial cells (HEp-2), lung epithelial cells (A549), lung fibroblasts (MRC-5) and macrophages (RAW 264.7), ultimately resulting in cellular damage and death.^60^,^61^,^62^ Together, these observations suggest that host-like CO_2_ concentrations play a critical role in the early stages of *Scedosporium* and *Lomentospora* pathogenesis by accelerating germination, promoting metabolic activation, enhancing hyphal development and facilitating tissue invasion (**Figure 3**), ultimately increasing their pathogenic potential in mammalian hosts.

## Limitations

This review has several limitations that should be acknowledged. As a narrative synthesis, it is subject to potential selection bias and may not comprehensively capture all available studies. In addition, considerable heterogeneity exists across the studies analyzed, including differences in CO_2_ concentrations, exposure durations, culture conditions, experimental models and fungal strains. This variability limits direct comparability between studies and may influence the consistency and generalizability of the conclusions drawn. Furthermore, a substantial proportion of the available evidence is derived from *in vitro* systems, which may not fully recapitulate the dynamic and complex conditions encountered within host environments, such as tissue-specific CO_2_ gradients, immune pressures and interactions with other microorganisms. The relative scarcity of *in vivo* and clinical data also constrains the ability to translate these findings into pathophysiological or therapeutic conditions fully. Finally, variability in methodological approaches and reporting standards across studies may further complicate the interpretation and integration of the data.

## Conclusion

The body of evidence reviewed here clearly indicates that pathogenic fungi belonging to the genera *Candida*, *Cryptococcus*, *Aspergillus* and *Scedosporium*/*Lomentospora* have evolved sophisticated systems to sense, interpret and respond to fluctuations in environmental CO_2_. Far from being a passive atmospheric component, CO_2_ acts as a biologically active host-derived signal that reshapes fungal physiology at multiple levels. Exposure to host-like CO_2_ concentrations influences key processes including morphogenetic transitions, biofilm formation, capsule production, sporulation, metabolic reprogramming and mitochondrial activity (**Table 1**). Importantly, CO_2_ also modulates antifungal susceptibility in a species-specific manner, either enhancing or reducing drug responsiveness depending on the organism and environmental condition. These adaptive responses have direct implications for virulence expression, tissue invasion, persistence within the host and ultimately clinical outcomes. Collectively, these findings highlight CO_2_ as a central environmental determinant in fungal pathobiology.

## Data Availability

*Not applicable.*
